# Successful intracranial response of lorlatinib after resistance with alectinib and brigatinib in patients with ALK‐positive lung adenocarcinoma: Implications of CNS penetration rate of brigatinib

**DOI:** 10.1111/1759-7714.15395

**Published:** 2024-06-24

**Authors:** Yuki Sato, Yoshiharu Sato, Kei Irie, Shigeki Nanjo, Shigeo Hara, Satoru Fujiwara, Keisuke Tomii

**Affiliations:** ^1^ Department of Respiratory Medicine Kobe City Medical Center General Hospital Kobe Japan; ^2^ DNA Chip Research Inc Tokyo Japan; ^3^ Department of Pharmaceutics Faculty of Pharmaceutical Science, Kobe Gakuin University Kobe Japan; ^4^ Department of Respiratory Medicine Kanazawa University Hospital Kanazawa Japan; ^5^ Department of Pathology Kobe City Medical Center General Hospital Kobe Japan; ^6^ Department of Neurology Kobe City Medical Center General Hospital Kobe Japan

**Keywords:** ALK, brigatinib, CNS penetration, lorlatinib, non‐small cell lung cancer

## Abstract

We present the case of a 34‐year‐old Japanese man with anaplastic lymphoma kinase (ALK)‐positive non‐small cell lung cancer and brain metastases. After central nervous system (CNS) disease progression with alecintib and brigatinib, treatment with lorlatinib resulted in a good intracranial response. In this case, we investigated brain penetration ratio of brigatinib using cerebrospinal fluid and paired serum samples, and the ratio was 0.012. Further, we investigated resistance mechanisms via next‐generation sequencing (NGS) using lung biopsy at lung cancer diagnosis and brain biopsy sample at progressive disease of brigatinib. No apparent resistance mechanism of known ALK resistance, such as *ALK* mutations, amplifications, epithelial‐mesenchymal transition (EMT) and bypass pathway activation were detected. Taken together, we speculate that the low CNS penetration rate of brigatinib confers CNS progression. Further studies are warranted to reveal the resistance mechanism and propose a treatment strategy for CNS progression in ALK‐positive patients.

## Graphical abstract

We present the case of a 34‐year‐old Japanese man with anaplastic lymphoma kinase (ALK)‐positive non‐small cell lung cancer and brain metastases. After central nervous system (CNS) disease progression with alecintib and brigatinib, treatment with lorlatinib resulted in a good intracranial response. In this case, we investigated brain penetration ratio of brigatinib using cerebrospinal fluid and paired serum samples, and the ratio was 0.012. We speculate that the low CNS penetration rate of brigatinib confers CNS progression.

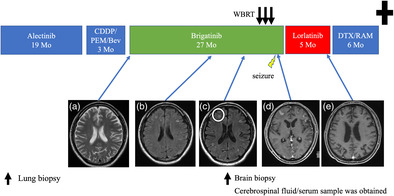

## INTRODUCTION

Patients with advanced non‐small cell lung cancer (NSCLC) harboring an anaplastic lymphoma kinase (ALK) rearrangement receive extended benefits from ALK tyrosine kinase inhibitors (TKIs). However, acquired resistance to ALK‐TKIs inevitably develops and is often mediated by the acquisition of secondary *ALK* mutations or amplifications. Other resistance mechanisms mediated by the activation of different bypass signaling pathways, epithelial‐to‐mesenchymal transition (EMT), histological transformation, and other epigenetic alterations have also been reported. Resistance implies the absence of ALK‐TKI activity, leading to poor outcomes; however, the underlying mechanisms have been poorly investigated, especially in central nervous system (CNS) lesions, owing to the difficulty in obtaining CNS tissue samples.

Here, we report a case of alectinib‐ and brigatinib‐resistant EML4‐ALK translocation‐positive lung cancer with brain metastasis. Notably, CNS resistance to alectinib and brigatinib was overcome by the subsequent administration of lorlatinib. In this case, we explored clinical samples of patients (cerebrospinal fluid/serum and lung/brain tissue). Through next‐generation sequencing (NGS) analysis, we investigated the CNS penetration rate of brigatinib and possible resistance mechanisms in CNS lesions.

## CASE REPORT

**FIGURE 1:**
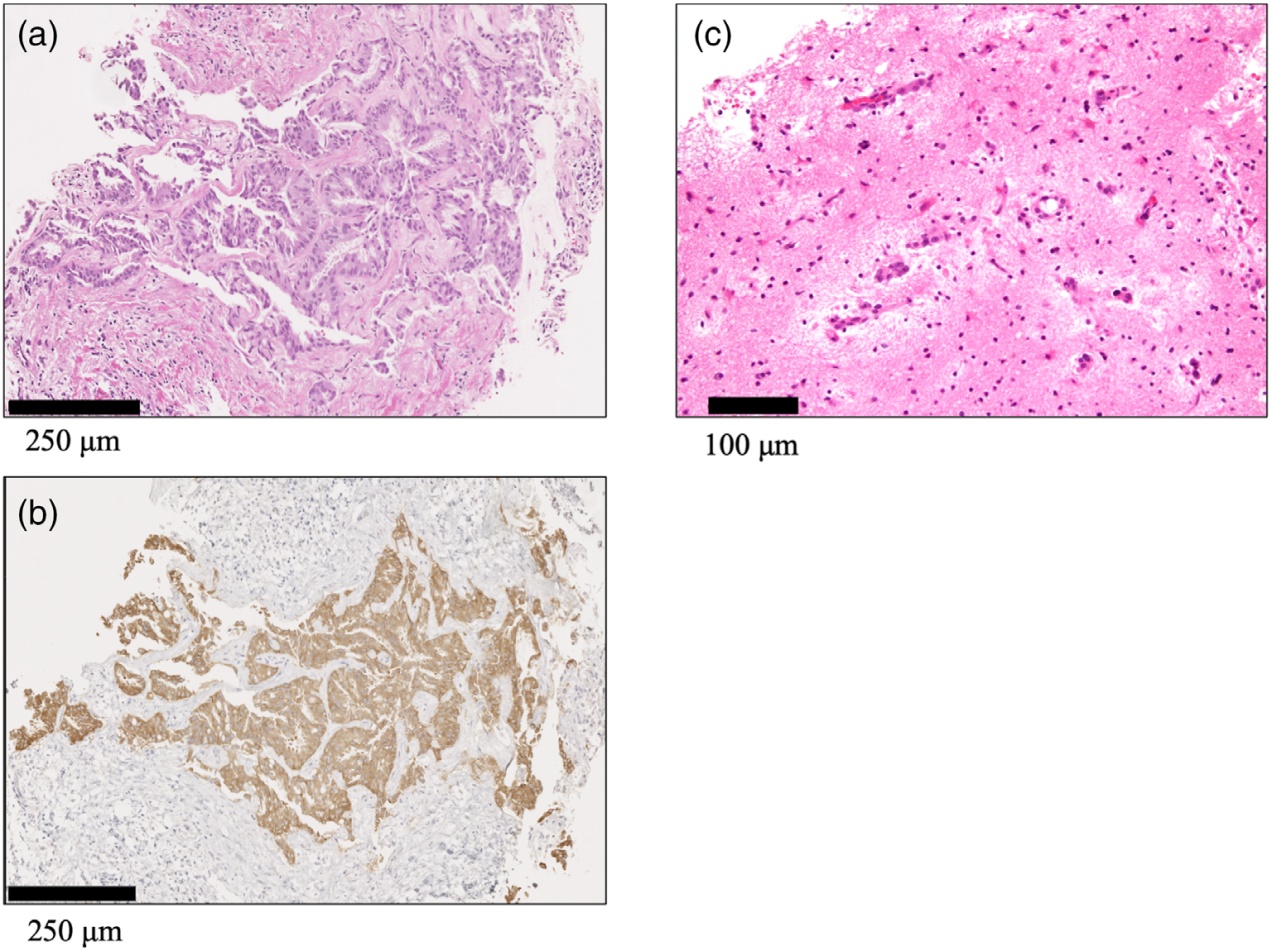
(a) Hematoxylin‐eosin (HE) staining of lung biopsy samples at initial diagnosis (×200). (b) Results of ALK immunohistochemistry (×200). (c) HE staining of brain biopsy samples at resistance to brigatinib (×200).

**FIGURE 2:**
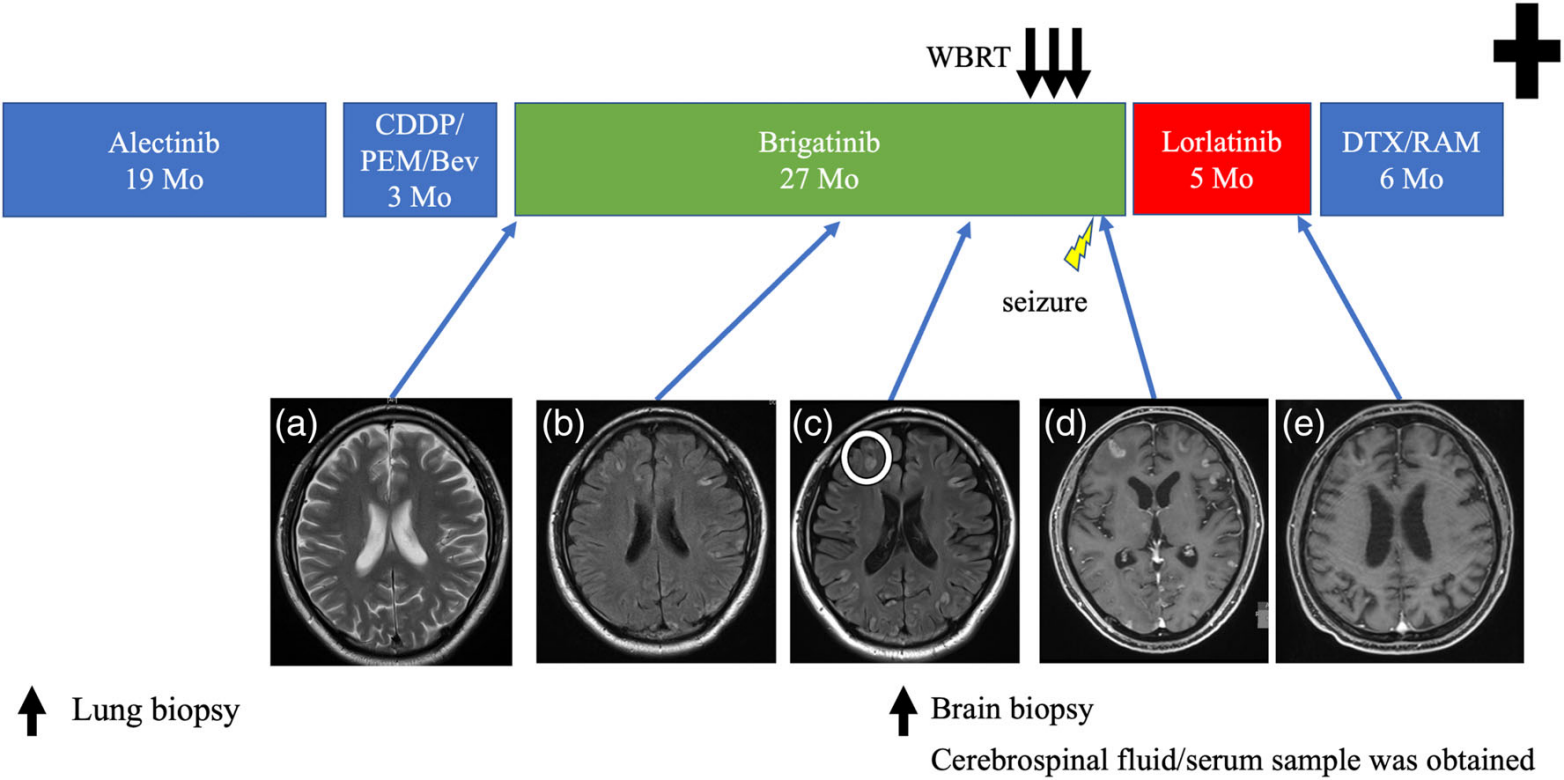
Treatment course and brain MRI images. (a) Disease progression of brain metastases following platinum doublet therapy with angiogenesis inhibitor. (b,c) Nodules with a slight contrast effect appeared and gradually progressed. A brain biopsy was performed from the right frontal lobe. (d) Brain metastases worsened despite whole‐brain radiation therapy. (e) After initiation of lorlatinib, the brain lesion improved. CDDP, cisplatin; Bev, bevacizumab; DTX, docetaxel; MRI, magnetic resonance imaging; PEM, pemetrexed; RAM, ramucirumab.

A 34‐year‐old man with no significant medical or smoking history was referred to our hospital with a history of a dry cough. Bronchoscopy revealed lung adenocarcinoma (cT1aN3M1a, cStage IVA, EML‐ALK translocation‐positive). Lung biopsy specimens are shown in Figure 1a,b (hematoxylin–eosin staining and ALK immunohistochemistry, antibody D5F3). Alectinib 600 mg was initiated as the first‐line treatment, and progressive disease was confirmed 19 months later. Subsequently, platinum plus an antiangiogenesis inhibitor was initiated as a second‐line treatment, but multiple brain metastases were identified on brain magnetic resonance imaging (MRI) after 3 months (Figure 2a). Brigatinib (starting dose of 90 mg, increased to 180 mg after 7 days of exposure) was introduced as a third‐line treatment. Although brigatinib achieved a partial response (Figure 2b), MRI detected multiple brain nodules 13 months after initiating brigatinib (Figure 2c). To distinguish brain metastasis from the adverse effects of ALK inhibitors, a brain biopsy revealed adenocarcinoma cells (Figure 1c). Therefore, the patient was diagnosed with an exacerbation of brain metastasis. Whole‐brain radiation therapy was administered, and brigatinib was continued; however, symptomatic epilepsy and CNS lesions worsened on MRI (Figure 2d). Brigatinib was then switched to lorlatinib (100 mg once daily). After 5 months, the metastatic lesions in the brain had significantly improved (Figure 2e). Because of the exacerbation of the extracranial lesions, the chemotherapy regimen was switched to a cytotoxic agent.

We investigated the resistance mechanism of brigatinib in the CNS using clinical samples, including cerebrospinal fluid (CSF) and serum collected at progressive disease of brigatinib and lung and brain biopsies at diagnosis/progressive disease of brigatinib. Our analysis revealed that the concentration of brigatinib in CSF was 13.23 ng/mL, whereas the total plasma concentration collected at the trough was 1039 ng/mL, as determined by liquid chromatography with tandem mass spectrometry (LC‐MS/MS).^1^ This resulted in a CSF‐to‐serum concentration ratio of 0.012 (Table 1). Subsequently, we performed RNA and next‐generation sequencing on the paired specimens (Oncomine Focus Assay and ION Ampliseq Comprehensive Cancer Panel, Thermo Fisher Scientific; detailed methods are shown in Supporting Information data S1). Our analysis revealed the presence of an ALK fusion array (variant 2) in exon20 in both samples, as shown in Figure S1a. We also analyzed the known mechanisms of resistance, such as secondary *ALK*‐mutation (Figure S1b), ALK‐amplification, bypass pathways, and the number of *MET* copies (Figure S1c); however, no apparent resistance mechanism was detected.

**TABLE 1:** Concentration of brigatinib and lorlatinib in CNS fluid and serum.

Compound	Brigatinib	Lorlatinib				
Patient	This case	Clinical trial no. 1	Clinical trial no. 2	Clinical trial no. 3	Clinical trial no. 4	Clinical trial no. 5
Total plasma concentration (ng/mL)	1039	12.7	384	457	311	165
CSF concentration (ng/mL)	13.23	2.64	125	101	81.8	38.1
CNS/plasma ratio (total)	0.012	0.208	0.326	0.221	0.263	0.231

Abbreviations: CNS, central nervous system; CSF, cerebrospinal fluid.

## DISCUSSION

In this report, we present a patient with ALK‐positive NSCLC who demonstrated an impressive CNS response to lorlatinib following resistance to alectinib and brigatinib. Lorlatinib, a third‐generation ALK‐TKI, has shown improved efficacy against CNS metastases. However, its effectiveness following resistance to second‐ or third‐generation ALK‐TKIs is poorly documented despite being a commonly encountered problem in clinical settings.

Generally, resistance mechanisms in CNS lesions are difficult to analyze because of the scarcity of tissue specimens. Our case report is unique in that we analyzed brain tissue and CSF samples from the patient. To the best of our knowledge, the CNS penetration rate of brigatinib has not been previously reported. Notably, the CNS penetration rate of brigatinib was 0.012. We did not evaluate CNS penetration rate of lorlatinib in this case as the patient did not provide informed consent. However, it was strikingly lower than that of lorlatinib (0.208–0.326) reported in subanalysis of phase I/II clinical trials (Table 1).^2^ As for alectinib, a previous phase I/II study demonstrated it to be approximately 0.7.^3^ In addition, we investigated the resistance mechanisms using NGS. Our findings revealed no emergence of secondary mutations (gatekeeper mutations), amplification of *ALK* fusion genes, or activation of bypass pathways independent of ALK.^4^ These findings suggest that the resistance to brigatinib observed in this patient may have been mediated by low delivery to CNS lesions. Considering the importance of drug delivery, switching to lorlatinib may be a promising treatment option for patients with brain metastases who experience symptom exacerbation during treatment with other ALK‐TKIs.

In conclusion, we present a case of successful treatment with lorlatinib following the progression of brigatinib and alectinib in a patient with *ALK*‐positive NSCLC with brain metastasis. The lower CNS penetration of ALK‐TKIs may have contributed to patient resistance, and lorlatinib may be a viable treatment strategy for patients with brain metastases after treatment with multiple ALK‐TKIs.

## AUTHOR CONTRIBUTIONS

**Yuki Sato:** Data curation, methodology, formal analysis, funding acquisition, investigation, resources, visualization, project administration, writing the original draft, writing the review and editing. **Yoshiharu Sato:** Data curation, methodology, formal analysis, investigation, visualization, writing, review and editing. **Kei Irie:** Data curation, methodology, formal analysis, investigation, visualization, writing, review and editing. **Shigeki Nanjo:** Data curation, methodology, formal analysis, investigation, writing – review and editing. **Hara Shigeo:** Data curation, methodology, formal analysis, investigation, visualization, writing, review and editing. **Satoru Fujiwara:** Data curation, methodology, formal analysis, investigation, visualization, writing, review and editing. **Keisuke Tomii:** Project administration, writing – review and editing.

## CONFLICT OF INTEREST STATEMENT

Dr Yuki Sato received personal fees from AstraZeneca, Chugai Pharmaceutical, MSD, Ono Pharmaceutical, Novartis, Pfizer, Taiho Pharmaceutical, Nippon Kayaku, Bristol Myers Squibb, Eli Lilly, Takeda, and Kyowa Kirin outside the submitted work. Dr Yoshiharu Sato is an employee of DNA Chip Research Inc., Tokyo, Japan. Dr Fujiwara received personal fees from Biogen Japan and Daiichi Sankyo outside the submitted work. Dr Tomii received honoraria from Astellas, AstraZeneca, Boehringer Ingelheim, Bristol Myers Squibb, Chugai Pharma, Daiichi Sankyo, Eli Lilly, GlaxoSmithKline, Kyorin, Kyowa Hakko Kirin, MSD, Nippon Kayaku, Novartis Pharma, Pfizer, Sanofi, Shionogi, Taiho Pharmaceutical, and Teijin Pharma, and has an advisory role at Eli Lilly outside the submitted work.

All remaining authors have declared no conflicts of interest.

## Supporting information


**Figure S1.** (a) RNA sequencing results of ALK sites showing *ALK* translocation (variant 2). (b) Sequence data of the G1202 site with no secondary *ALK* mutations detected. (c) Copy number analysis of all genes (a) and *MET* (b) using initial lung cancer diagnosis specimen, and all genes (c) and *MET* (d) using specimen at progressive disease of brigatinib, which revealed no copy number gain in *MET* at resistance to brigatinib (b vs. d, *p*‐value: 0.17).

**Data S1:** Supporting information.

## Data Availability

The corresponding author declares full access to all data in the study and takes responsibility for the integrity of the data and the accuracy of the data analysis.
