# Supplementary figures and images for: Successful intracranial response of lorlatinib after resistance with alectinib and brigatinib in patients with ALK‐positive lung adenocarcinoma: Implications of CNS penetration rate of brigatinib
Source: Thorac Cancer. 2024 Jun 24;15(23):1772–5. doi: 10.1111/1759-7714.15395 (PMC11320077; doi:10.1111/1759-7714.15395)

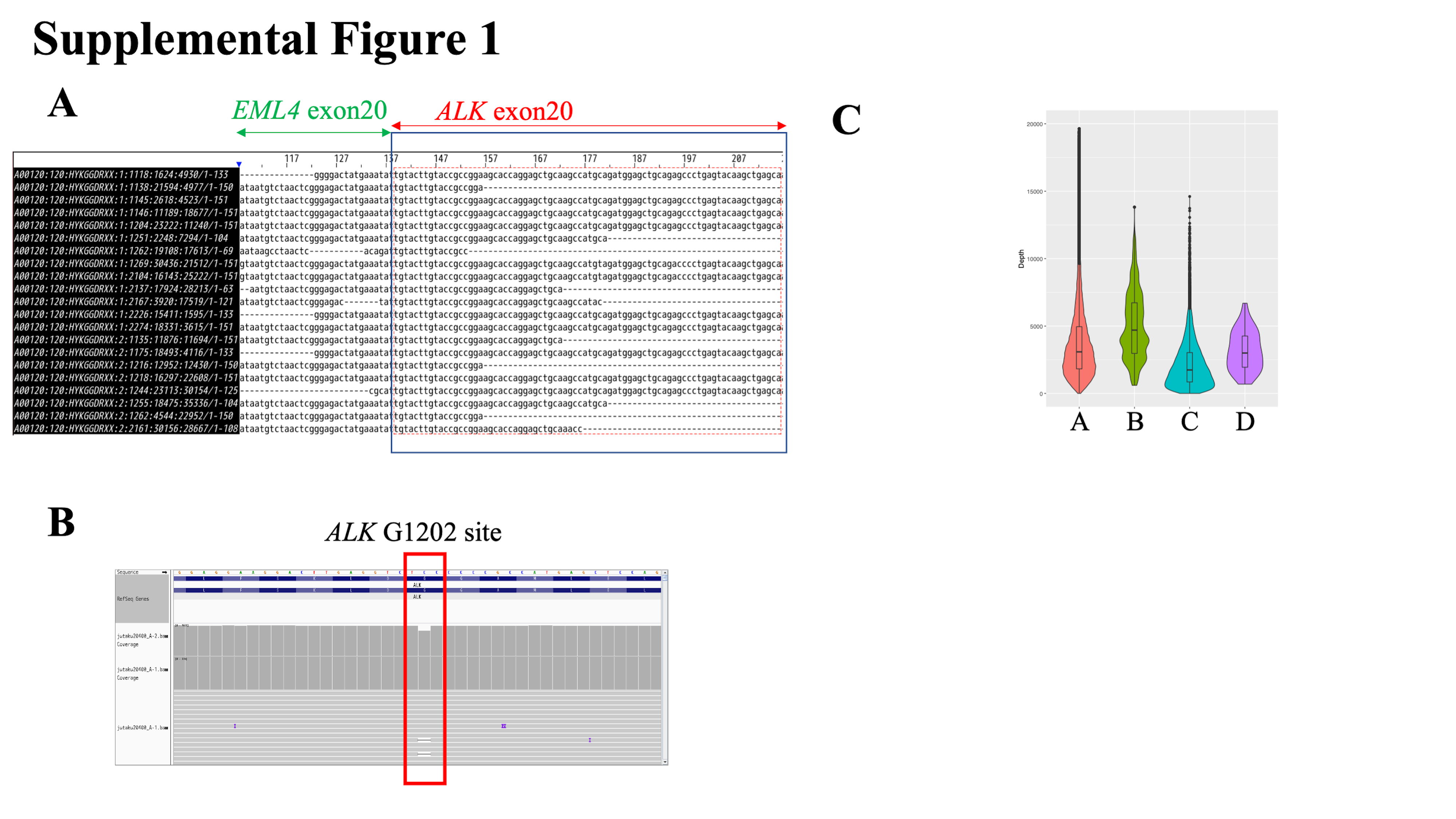

Supplement: Supplementary file 1 — Figure S1. (a) RNA sequencing results of ALK sites showing ALK translocation (variant 2). (b) Sequence data of the G1202 site with no secondary ALK mutations detected. (c) Copy number analysis of all genes (a) and MET (b) using initial lung cancer diagnosis specimen, and all genes (c) and MET (d) using specimen at progressive disease of brigatinib, which revealed no copy number gain in MET at resistance to brigatinib (b vs. d, p‐value: 0.17). [file TCA-15-1772-s002.tiff]
