# Supplementary material for: Successful intracranial response of lorlatinib after resistance with alectinib and brigatinib in patients with ALK‐positive lung adenocarcinoma: Implications of CNS penetration rate of brigatinib
Source: Thorac Cancer. 2024 Jun 24;15(23):1772–5. doi: 10.1111/1759-7714.15395 (PMC11320077; doi:10.1111/1759-7714.15395)
Supplement: Supplementary file 2 — Data S1: Supporting information. [file TCA-15-1772-s001.docx]

**Supplementary Material**

**Materials and Methods**

**Sample preparation**

Genomic DNA and total RNA were isolated from each FFPE sample using the Maxwell RSC DNA FFPE and Maxwell RSC RNA FFPE Kits (*Promega, Madison, WI, USA*). The integrity and quantity of total RNA were measured using the Agilent 2100 Bioanalyzer RNA 6000 Pico Kit (*Agilent Technologies, Santa Clara, CA*). Genomic DNA quality was assessed using a Genomic DNA ScreenTape Assay on a TapeStation 2200 instrument (*Agilent Technologies, Santa Clara, CA*).

**DNA Panel Analysis: Targeted Enrichment, Sequencing, Data Analysis**

Target regions from 409 oncogenes and tumor suppressor genes were enriched by multiplex PCR using the Ion AmpliSeq^TM^ Comprehensive Cancer Panel (*Thermo Fisher Scientific*), according to the manufacturer’s instructions using 65 ng of gDNA with each. PCR amplicon-based sequencing libraries were constructed using Ion AmpliSeq Library Kit 2.0 (*Thermo Fisher Scientific*). Library quality was assessed using a High-Sensitivity D1000 ScreenTape assay (*Agilent Technologies, Inc.*) on a TapeStation 2200. Multiplex barcoded and equally pooled libraries were sequenced using the 540 chip by Ion GeneStudio S5 System (*Thermo Fisher Scientific*). Variants among the sequence results obtained from the Ion S5 system were detected using the Torrent Variant Caller of the Ion Reporter Software (v5.10; *Thermo Fisher Scientific*; https://ionreporter.thermofisher.com/ir/). Tumor-normal matched-pair analysis was performed to detect somatic mutations (SNVs and CNVs). The default variant filter chain was used to filter detected variants.

**RNASeq: Library Preparation and Sequencing**

Sequencing libraries were constructed using the SMART-Seq Stranded Kit (*Clontech*) with the SMARTer RNA Unique Dual Index Kit according to the manufacturer’s protocols. Library quality was assessed using Agilent 2200 TapeStation High Sensitivity D1000 (*Agilent Technologies, Santa Clara, CA*). The pooled libraries of the samples were sequenced using NovaSeq 6000 (*Illumina, Inc., San Diego, CA*) in 150-base-pair (bp) paired-end reads.

**RNASeq:Data Analysis**

Sequencing adaptors, low-quality reads, and bases were trimmed with the Trimmomatic-0.38 tool^1^. Sequence reads were aligned to the human reference genome (hg38) using STAR 2.7.8a^2^.

The aligned reads were subjected to downstream analyses using StrandNGS 3.2 software (*Agilent Technologies, Santa Clara, CA*). The read counts allocated to each gene and transcript (Ensembl Genes 2016.12.01) were quantified using the transcripts per million (TPM) method^3^.

**References**

1. Bolger AM, Lohse M, Usadel B. Trimmomatic: a flexible trimmer for Illumina sequence data. Bioinformatics 2014;30:2114-20.

2. Dobin A, Davis CA, Schlesinger F et al. STAR: ultrafast universal RNA-seq aligner. Bioinformatics 2013;29:15-21.

3. Wagner GP, Kin K, Lynch VJ. Measurement of mRNA abundance using RNA-seq data: RPKM measure is inconsistent among samples. Theory Biosci 2012;131:281-5.
